# Dr Sabina Strich BCh, DM, MRCPath, MRCP, FRCPsych: Formerly Honorary Consultant Neuropathologist, The Bethlem Royal andThe Maudsley hospitals, Reader in Neuropathology, Institute of Psychiatry and Consultant Child and Adolescent Psychiatrist, Croydon Child Guidance Clinic

**DOI:** 10.1192/pb.bp.116.054320

**Published:** 2016-10

**Authors:** Peter Agulnik

**Figure:**
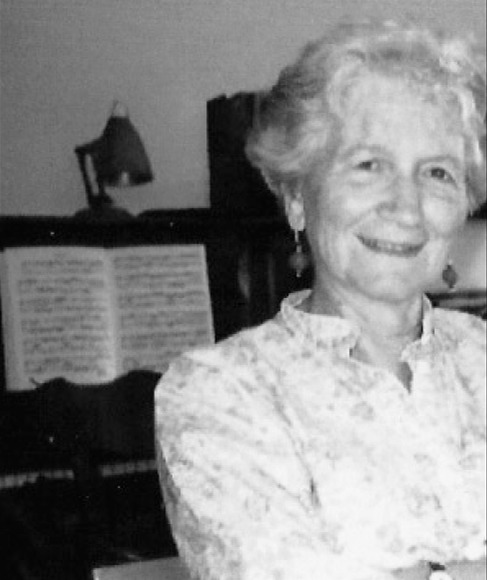


Dr Sabina Strich, who died aged 90 on 23 May 2015, made pioneering discoveries in the 1950s on the effects of head trauma on the brain. While carrying out histopathological studies with Peter Daniel in Oxford, she identified characteristic changes to brain cells following severe head injuries. She showed that shearing injuries, arising as a result of rapid acceleration or deceleration at the time of trauma, produced diffuse degeneration of the cerebral white matter^1^. The histopathological changes she described later became known as diffuse axonal injury (DAI) or Sabina Strich syndrome. She claimed that cellular changes of this type were the cause of later dementia. In 1957, shortly after the publication of this highly original work, she followed Peter Daniel to the Institute of Psychiatry, London where she continued her work on the histopathology of the brain, being appointed senior lecturer and subsequently Reader in Neuropathology.

However, in 1973, she decided to change specialties and retrain as a child psychiatrist and psychotherapist. In many ways a private person, she volunteered little on what led to that change. Even to close friends, she only revealed that she was more interested in the mind than the brain. One conjectures an epiphany associated with having undergone a Freudian and then a Jungian analysis, linked perhaps to her family's cultural heritage and lifelong interest in ideas.

Two years after becoming an honorary clinical assistant at the Belgrave Hospital for Children, she moved on to senior registrar posts, first at King's College Hospital and then at The Royal London Hospital. In 1977 she was appointed consultant psychiatrist to the Croydon Child Guidance Clinic. Here she particularly enjoyed working with families and joined The Association of Family Therapists. She was noted for her friendly but idiosyncratic clinical style, often forthright, but touched with a warm humour. In addition to the benefits deriving from her own psychoanalysis, she obtained experience of group work during the early years of the Institute of Group Analysis. Later, as a member, she was actively involved in training group analysts on courses as far apart as Manchester and Heidelberg. By the time of her retirement in 1984, she recalled with some pleasure that she had attained membership of the two Royal Colleges devoted to medicine and pathology, and fellowship of the Royal College of Psychiatrists.

Sabina Strich was born in Munich into an intellectual Jewish family of private means. An older sister died when 9 years old. Her younger sister moved and brought up a family in the United States, where Sabina was a frequent visitor. With the rise of the Nazi party the family fled Germany, settling in Cambridge, where she attended The Perse School. In 1943 she won a scholarship to study medicine at Lady Margaret Hall, University of Oxford, gaining a degree in physiology before embarking on clinical studies as one of the earliest clinical students of the Oxford Medical School at the Radcliffe Infirmary. She qualified BM BCh in 1949. Following house posts in medicine and surgery in Oxford and Swansea, in 1951 she was awarded a Medical Research Council (MRC) scholarship for training in research methods, initially at Manchester and then in Professor Sir Hugh Cairns' surgical department at Oxford. Here she obtained a personal MRC grant to study the neuropathology of severe head injuries with Peter Daniel.

After her retirement she returned to Oxford, in many ways her spiritual home. She bought a riverside apartment and set about creating a new and active life. She loved companioned walking and travelling far afield. Not ready to give up psychotherapy, she approached the Isis Centre, an open access NHS counselling service whose small number of core staff was augmented by associate counsellors. Sabina generously volunteered in a number of roles – such as a consultant in weekly case discussion groups – and in a range of educational events. She ran a client group and an experiential group for nurses studying psychodynamic practice, saw couples and did some family work. She was especially valued as a supervisor in all these modalities as well as in individual work. She developed a small private practice and took active roles within the Oxford Psychotherapy Society, of which she was a founding member. She spoke on a number of occasions, as well as submitting articles and commentaries.

To all her professional activities she brought a distinctive ‘Sabinoid style’. As a supervisor, her comments tended to be few and authoritative, but never critical, thus facilitating an ambience which encouraged others to develop their own ideas. After 11 years she left the Isis Centre, but remained linked with her many friends in the psychotherapy world.

Towards the end of this period, she developed a new interest. Having been led to believe as a child that she had no artistic abilities, she experimented with clay and was surprised and delighted to discover that distinctive, often quirky figures, with vitality and movement, emerged from her fingertips. Encouraged, she took lessons in sculpture and soon became an accomplished sculptress, exhibiting her work in local private exhibitions. Maybe it was in this creative work that her belief in the unconscious, her self-assurance and above all her empathic interest in people came to the fore.

Recognising mild cognitive impairment, in her 80th year she moved to a pleasant apartment in a retirement complex. Art exhibitions, theatre and music played an important part in her life; she regularly attended concerts and read widely. Always fond of poetry, she published a small book of her own verse, with poems which were short, powerful and laced with dry humour.

She found increasing deafness and lapses in memory deeply frustrating but – typically – Sabina responded by forming a group for the elderly with early dementia. She was active in maintaining her interests in medicine, religion, philosophy and psychoanalysis, attending a seminar in the last week of her life. Ever a realist, and having contentedly reached 90 years, she was reconciled to her own death and died after a short illness.
